# Mechanical complications in patients with ST-segment elevation myocardial infarction: A single centre experience

**DOI:** 10.1371/journal.pone.0209502

**Published:** 2019-02-22

**Authors:** Jonas Lanz, Dörte Wyss, Lorenz Räber, Stefan Stortecky, Lukas Hunziker, Stefan Blöchlinger, David Reineke, Lars Englberger, Thomas Zanchin, Marco Valgimigli, Dik Heg, Stephan Windecker, Thomas Pilgrim

**Affiliations:** 1 Department of Cardiology, Swiss Cardiovascular Center, University Hospital Bern, Bern, Switzerland; 2 Department of Cardiovascular Surgery, Swiss Cardiovascular Center, University Hospital Bern, Bern, Switzerland; 3 Clinical Trials Unit, University of Bern, Bern, Switzerland; 4 Institute of Social and Preventive Medicine, University of Bern, Bern, Switzerland; Klinikum Region Hannover GmbH, GERMANY

## Abstract

**Background:**

The study aims to assess characteristics and outcomes of patients suffering a mechanical complication (MC) after ST-segment elevation myocardial infarction (STEMI) in a contemporary cohort of patients in the percutaneous coronary intervention era.

**Methods and results:**

This retrospective single-center cohort study encompasses 2508 patients admitted with STEMI between March 9, 2009 and June 30, 2014. A total of 26 patients (1.1%) suffered a mechanical complication: ventricular septal rupture (VSR) in 17, ventricular free wall rupture (VFWR) in 2, a combination of VSD and VFWR in 2, and papillary muscle rupture (PMR) in 5 patients. Older age (74.5 ± 10.4 years versus 63.9 ± 13.1 years, p < 0.001), female sex (42.3% versus 23.3%, p = 0.034), and a longer latency period between symptom onset and angiography (> 24h: 42.3% versus 16.2%, p = 0.002) were more frequent among patients with MC as compared to patients without MC. The majority of MC patients had multivessel disease (77%) and presented in cardiogenic shock (Killip class IV: 73.1%). Nine patients (7 VSR, 2 VFWR & VSR) were treated conservatively and died. Out of the remaining 10 VSR patients, four underwent surgery, three underwent implantation of an occluder device, and another three patients had surgical repair following occluder device implantation. All patients with isolated VFWR and PMR underwent emergency surgery. At 30 days, mortality for VSR, VFWR, VFWR & VSR and PMR amounted to 71%, 50%, 100% and 0%, respectively.

**Conclusions:**

Despite advances in the management of STEMI patients, mortality of mechanical complications stays considerable in this contemporary cohort. Older age, female sex, and a prolonged latency period between symptom onset and angiography are associated with the occurrence of these complications.

## Introduction

Papillary muscle rupture, ventricular septal rupture and free wall rupture complicating myocardial infarction are rare but devastating sequelae of myocardial necrosis. Although the incidence of mechanical complications has decreased owing to the development of reperfusion and adjunct medical therapies, these adverse events still go along with an exceptionally high mortality rate and constitute one of the major causes of death in the early phase after myocardial infarction. [1–6]

Most available data describing the frequency and characteristics of mechanical complications stem from the early reperfusion era evaluating fibrinolytic therapy. [1–4] The objective of the present study is to assess characteristics and outcomes of patients with a mechanical complication (MC) after ST-elevation myocardial infarction (STEMI) in a contemporary cohort of consecutive patients in the era of primary percutaneous coronary intervention.

## Methods

### Study population and data sources

This retrospective single-center cohort study encompasses all patients, who suffered a MC during the course of STEMI, and were admitted to the University Hospital in Bern, Switzerland, a large tertiary cardiology center, between March 9, 2009 and June 30, 2014. The Cardiobase Bern PCI Registry collects clinical, procedural and outcome data of all consecutive patients undergoing percutaneous coronary intervention (PCI) at the University Hospital of Bern, Switzerland. The registry (NCT02241291) has been approved by the Ethics Commission of the Canton of Bern, Switzerland (KEK 137/14). Patients provided written informed consent, in case a patient died peri- or post-procedurally, the request for written informed consent was waived by the responsible ethics committee in order to avoid the introduction of a substantial selection bias. All medical records of patients with a MC were independently retrieved, consulted and carefully reviewed by two investigators (JL, DW) to confirm the complication, capture patient characteristics and document the individual management.

### Definitions and follow-up

STEMI was defined on the baseline ECG as ST-segment elevation of at least 1 mm in two consecutive extremity leads or of at least 2 mm in two consecutive precordial leads, or the presence of a new left bundle branch block in combination with typical symptoms. The following three major entities were considered as mechanical complications: rupture of the left ventricular free wall, rupture of the papillary muscle, and ventricular septal rupture. Only the first event per non-lethal event type was counted per patient during follow-up. Renal failure was defined as a glomerular filtration rate < 60 ml/min/1,73 m^2^ based on the Cockcroft-Gault formula, anemia was defined as a hemoglobin value < 130 g/L for men and < 120 g/L for women and thrombocytopenia as a thrombocyte count < 150 G/l. Outcomes were assessed at hospital discharge and 1-year telephone follow-up. The Clinical Trials Unit and the Department of Cardiology at Bern University Hospital had the responsibility of data monitoring and storing for the registry.

### Statistical analysis

Patient characteristics, comorbidities, laboratory values and procedural characteristics are presented as counts (percentage) for categorical variables and mean (± standard deviation (SD)) or median (inter-quartile range (IQR)) for continuous ones depending on data distribution. P-values were derived from unpaired t-tests or Mann-Whitney U-tests for comparisons of continuous data and chi-square or Fisher's exact tests for categorical variables. Failure curves contrasting mortality of patients with and without mechanical complications were generated by means of the Kaplan Meier method and compared using Cox proportional hazard regression.

## Results

**Fig 1:**
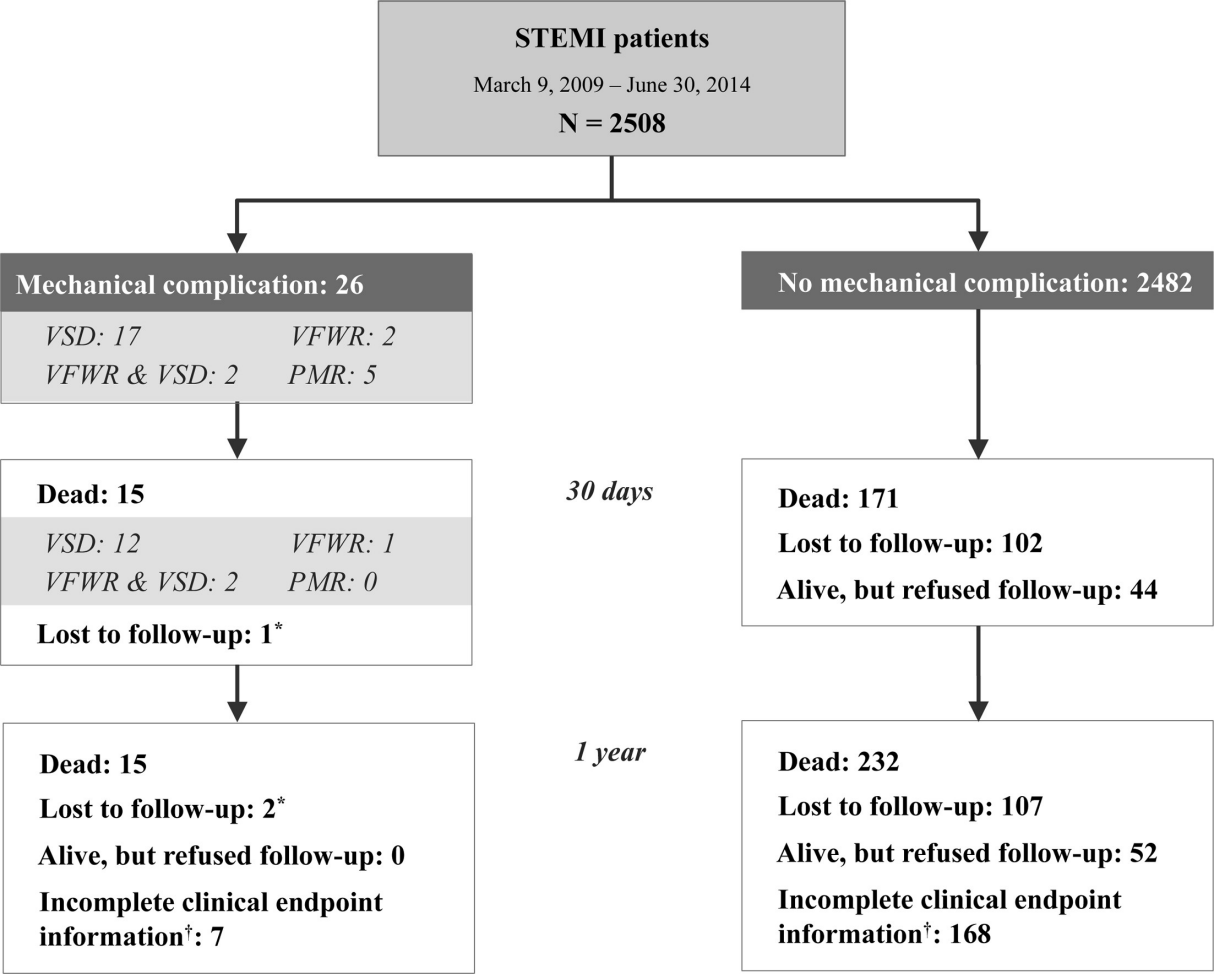
Study flowchart. 2508 patients with ST-elevation myocardial infarction (STEMI) admitted for percutaneous coronary intervention at the University Hospital Bern between March 9, 2009 and June 30, 2014. 26 suffered a mechanical complication. STEMI, ST-elevation myocardial infarction; VSD, ventricular septal defect; VFWR, ventricular free wall rupture; PMR, papillary muscle rupture; * first patient lost to follow-up at day 26, second one at day 358; ^**†**^ regarding non-lethal clinical endpoints.

A total of 2508 patients were admitted to Bern University Hospital, Switzerland with STEMI between March 9, 2009 and June 30, 2014. Twenty-six (1.1%) of these patients suffered a mechanical complication (MC). During 1-year follow, two patients were lost to follow-up in the MC, and 107 in the no-MC group (Fig 1).

Patients suffering a MC had a mean age of 74.5 years and were approximately ten years older than those without MC (Table 1). The proportion of women (42.3% versus 23.3%, p = 0.034) as well as the proportion of patients with renal failure on admission (66.7% versus 18.7%, p <0.001) was significantly higher in the MC group compared to the no-MC group. Left ventricular ejection fraction was slightly reduced in both groups (Table 1). Patients in the MC group frequently suffered from anemia (48%) or thrombocytopenia (20.8%) upon presentation. The majority of patients admitted with a MC (73.1%) were in cardiogenic shock (Killip class IV), whereas most patients (71.9%) without MC presented without signs of heart failure (Killip class I) (Table 1).

**Table 1:** Baseline characteristics.

	Mechanical complication N = 26	No mechanical complication N = 2482	p-value
**Demographics**			
Age (years)	n = 26, 74.5 ± 10.4	n = 2482, 63.9 ± 13.1	< 0.001
Female gender	n = 26, 11 (42.3%)	n = 2482, 578 (23.3%)	0.034
**Comorbidities, lifestyle and medical history**			
Atrial fibrillation/flutter	n = 25, 2 (8.0%)	n = 1137, 65 (5.7%)	0.651
BMI (kg/m^2^)	n = 25, 26.3 ± 4.4	n = 2271, 27.1 ± 4.4	0.371
Cerebrovascular accident	n = 26, 2 (7.7%)	n = 2447, 95 (3.9%)	0.272
COPD	n = 26, 1 (3.8%)	n = 2447, 100 (4.1%)	1.000
Current Smoker	n = 25, 8 (32.0%)	n = 2402, 980 (40.8%)	0.419
Diabetes mellitus	n = 26, 7 (26.9%)	n = 2453, 384 (15.7%)	0.169
Gastrointestinal bleeding	n = 26, 0 (0.0%)	n = 2447, 35 (1.4%)	1.000
Hyperlipidemia	n = 26, 10 (38.5%)	n = 2428, 1118 (46.0%)	0.554
Hypertension	n = 26, 16 (61.5%)	n = 2441, 1313 (53.8%)	0.554
Peripheral artery disease	n = 26, 2 (7.7%)	n = 2442, 95 (3.9%)	0.272
Myocardial infarction	n = 26, 1 (3.8%)	n = 2447, 195 (8.0%)	0.717
PCI	n = 26, 1 (3.8%)	n = 2447, 220 (9.0%)	0.724
CABG	n = 26, 1 (3.8%)	n = 2449, 71 (2.9%)	0.538
Renal failure	n = 24, 16 (66.7%)	n = 1203, 225 (18.7%)	< 0.001
**Echocardiography**			
LVEF (%)	n = 26, 46.9 ± 14.4	n = 2407, 45.4 ± 11.9	0.506
**Laboratory evaluation**			
Anemia	n = 25, 12 (48.0%)	n = 1192, 207 (17.4%)	< 0.001
Thrombocytopenia	n = 24, 5 (20.8%)	n = 1184, 12 (1.0%)	< 0.001
**Clinical presentation**			
Congestive heart failure	n = 26	n = 2471	< 0.001*
Killip I	2 (7.7%)	1776 (71.9%)	< 0.001
Killip II	4 (15.4%)	358 (14.5%)	0.783
Killip III	1 (3.8%)	91 (3.7%)	1.000
Killip IV	19 (73.1%)	246 (10.0%)	< 0.001

The number of patients for whom data for the corresponding variable was available (denominator) is reported (n), followed by the number of patients fulfilling the criterion (counts (%)) for categorical variables or means ± standard deviation for continuous ones. P-values are derived from Fisher's exact or chi-square-tests for categorical and unpaired t-tests for continuous data. BMI, body mass index. COPD, chronic obstructive pulmonary disease. PCI, percutaneous coronary intervention. CABG, coronary artery bypass grafting. LVEF, left ventricular ejection fraction.

* derived from chi-square -test comparing all categories.

In 50% of the patients with a MC PCI was not attempted or not completed (Table 2). In the MC group the time from symptom onset to balloon inflation or diagnostic angiography amounted to more than 24 hours in 42.3%, and only two patients (7.7%) underwent PCI within 6 hours, whereas almost half (48.2%) of the STEMI patients without mechanical complication were treated within 6 hours of symptom onset (Table 2). Only two patients had a history of PCI or CABG, for the remaining the STEMI leading to the MC was the first manifestation of coronary artery disease (Table 1). The right coronary artery was the most frequent culprit vessel in MC patients (13 (50%)), and multivessel disease was present in 77% (S1 Table). There was a significant difference in the proportion of patients with TIMI flow 0 or I between the MC group (82.4%) and the no-MC group (52.4%) (p = 0.014). At the end of coronary angiography TIMI flow III was documented in 82.4% of the culprit lesions in the MC group and achieved in 94.7% in STEMI patients without mechanical complications (Table 2). Circulatory support by means of intra-aortic balloon counter-pulsation (53.8% versus 4.6%, p < 0.001) or continuous vasopressor infusion (61.5% versus 9%, p < 0.001) was more frequently used in patients with MC compared to those without. Almost all patients with STEMI received a loading dose of a P2Y12 inhibitor (97.6%), whereas GPIIb/IIIa inhibitor use differed between groups (7.7% in the MC versus 22.6% in the no-MC group (Table 2)).

**Table 2:** Coronary angiography: Lesion and procedural characteristics.

	Mechanical complication N = 26	No mechanical complication N = 2482	p-value
**Latency period**			
Symptom onset to balloon inflation/angiography*	n = 26	n = 2466	< 0.001^†^
0–6 hours	2 (7.7%)	1189 (48.2%)	< 0.001
6–12 hours	5 (19.2%)	321 (13.0%)	0.374
12–24 hours	6 (23.1%)	179 (7.3%)	0.010
<24 hours^‡^	2 (7.7%)	378 (15.3%)	0.412
>24 hours	11 (42.3%)	399 (16.2%)	0.002
**PCI**			
PCI performed & completed	13 (50.0%)	2482 (100.0%)^§^	< 0.001
Number of lesions treated	1.3 ± 0.9	1.6 ± 0.9	0.265
Vessels treated	n = 17,	n = 3918,	0.478^†^
LM	0 (0.0%)	90 (2.3%)	1.000
LAD	4 (23.5%)	1665 (42.5%)	0.142
LCX	4 (23.5%)	664 (16.9%)	0.513
RCA	9 (52.9%)	1465 (37.4%)	0.213
Bypass graft	0 (0.0%)	34 (0.9%)	1.000
Multivessel treatment	n = 13, 2 (15.4%)	n = 2482, 461 (18.6%)	1.000
Total occlusion	n = 17, 11 (64.7%)	n = 3884, 1716 (44.2%)	0.140
Pre TIMI flow	n = 17,	n = 3843,	0.047^†^
0 or 1	14 (82.4%)	2013 (52.4%)	0.014
2	1 (5.9%)	650 (16.9%)	0.336
3	2 (11.8%)	1180 (30.7%)	0.115
Post TIMI flow	n = 17,	n = 3857,	0.008^†^
0 or 1	0 (0.0%)	65 (1.7%)	1.000
2	3 (17.6%)	140 (3.6%)	0.023
3	14 (82.4%)	3652 (94.7%)	0.059
Any drug-eluting stent	n = 14, 13 (92.9%)	n = 3618, 3248 (89.8%)	1.000
Any bare-metal stent	n = 14, 1 (7.1%)	n = 3618, 378 (10.4%)	1.000
Total stent length (mm)	n = 14, 30.7 ± 14.2	n = 3617, 27.7 ± 15.8	0.480
**Circulatory support**			
IABP	n = 26, 14 (53.8%)	n = 2481, 115 (4.6%)	< 0.001
Percutaneous LVAD	n = 26, 0 (0.0%)	n = 2145, 18 (0.8%)	1.000
Vasopressors	n = 26, 16 (61.5%)	n = 2479, 224 (9.0%)	< 0.001
**Peri-procedural medication**			
Unfractionated Heparin	n = 13, 12 (92.3%)	n = 2479, 2442 (98.5%)	0.181
LMWH	n = 13, 1 (7.7%)	n = 2478, 74 (3.0%)	0.329
GP IIb/IIIa	n = 13, 1 (7.7%)	n = 2478, 561 (22.6%)	0.320
P2Y12 loading dose	n = 13, 12 (92.3%)	n = 2482, 2423 (97.6%)	0.272

Depicted are means (± SD) or counts (%) with p-values derived from unpaired t-tests for continuous and Fisher's exact or chi-square-tests for categorical data. PCI, percutaneous coronary intervention. LM, left main. LAD, left anterior descending. LCX, left circumflex. RCA, right coronary artery. TIMI, Thrombolysis In Myocardial Infarction. IABP, intra-aortic balloon pump. LVAD, left ventricular assist device. LMWH, low molecular weight heparin. GP, Glycoprotein.

* time to angiography for patients without PCI.

^†^ derived from chi-square-test comparing all categories.

^‡^ exact onset time unknown.

^§^ in the registry only STEMI patients undergoing PCI are captured.

Ventricular septal ruptures (VSR), two in combination with free wall rupture, accounted for the most frequent type of mechanical complication (17 of 26) in our cohort (Table 3). Seven of these patients were treated conservatively and died (median time to death: 0 days, IQR: 0–1 days). In six VSR patients an occluder device was implanted; three of them died within days due to residual shunt and persistent cardiogenic shock and the remaining three required surgical reconstruction. Overall, seven VSR patients underwent cardiac surgery; all but two survived beyond 30 days.

**Table 3:** Mechanical complications: Management and 30-day mortality.

	N (%)	Management			30-day mortality			
		Conservative	Inter-ventional	Surgery	N (%)	Conservative	Inter-ventional	Surgery
**All**	26	9 (35%)	3^†^ (12%)	14^†^ (54%)	15 (58%)	9 (100%)	3^†^ (100%)	3^†^ (21%)
**Type:**								
Ventricular septal rupture (VSR)	17 (65.4%)	7* (41%)	3^†^ (18%)	7^†^ (41%)	12 (71%)	7 (100%)	3^†^ (100%)	2^†^ (29%)
Ventricular free wall rupture (VFWR)	2 (7.7%)	0	0	2 (100%)	1 (50%)	0	0	1 (50%)
VSR and VFWR	2 (7.7%)	2 (100%)	0	0	2 (100%)	2 (100%)	0	0
Papillary muscle rupture (PMR)	5 (19.2%)	0	0	5 (100%)	0 (0%)	0	0	0 (0%)

Depicted are counts (percentages). The percentages shown for the management categories refer to the total number of patients pertaining to the specific complication types. The reported percentages of patients dying within 30 days refer to the number of patients in the different management categories stratified by complication type. N, number.

* In two patients the attempt to implant an occluder device was unsuccessful.

^†^ Three patients, who received an occluder device, but consecutively underwent surgery, are assigned to the surgical group and not included in the interventional group; two of them survived, one died.

The two patients with isolated ventricular free wall rupture (VFWR) underwent emergency surgery; one of them died within 30 days. Two patients with VSR and additional VFWR succumbed to their mechanical complication on the day of admission. All five patients with papillary muscle rupture (PMR) underwent surgical mitral valve replacement and survived.

**Fig 2:**
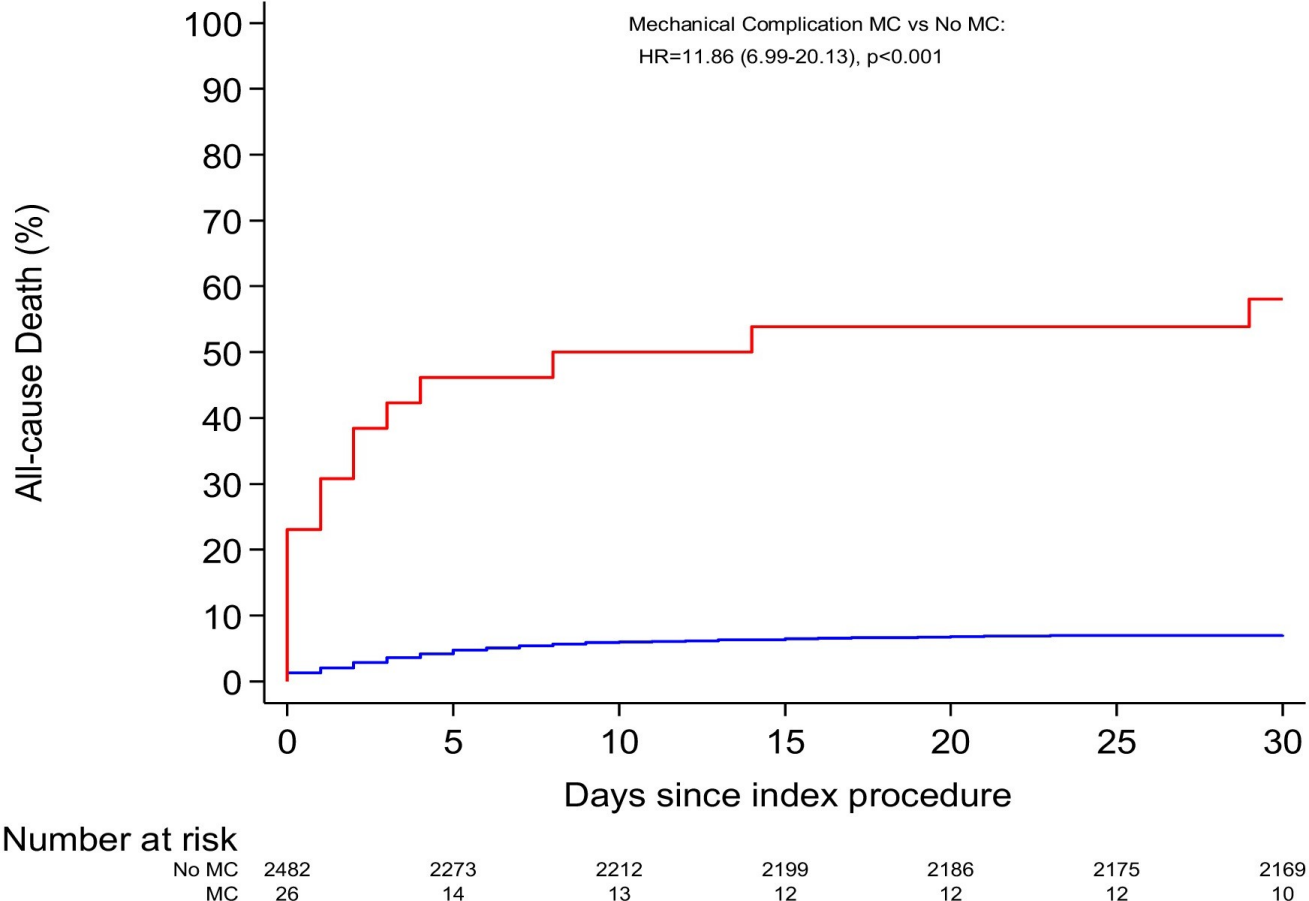
Survival curves. Depicted are survival curves for all-cause mortality based on the product-limit estimator comparing patients with and patients without a mechanical complication in the context of an ST-elevation myocardial infarction.

If a MC was managed surgically, the surgery was generally conducted promptly after diagnosis of the MC (median time to surgery: 0.5 days (IQR 0–1 day)). Overall 30-day mortality in the MC group amounted to 58%, in the no-MC group to 7%. The ratio contrasting the hazard rate of death at 30 days in the MC to the no-MC group amounted to 11.86 (95%-confidence interval (95%-CI): 6.99–20.13, p <0.001)) (Fig 2). Associations between patient characteristics and mortality based on univariate Cox regression analysis in the total STEMI cohort are presented in S2 Table. None of the MC patients, for whom complete follow-up data was available and who survived beyond 30 days, died up to one-year follow-up. Two of the MC patients experienced a stroke during the one-year follow-up period (HR comparing MC to no-MC: 14.14, 95% CI 3.32–60.29).

## Discussion

In the present cohort study approximately one percent of the patients admitted with a STEMI suffered a MC. Patients with MC were older and more likely to be females as compared to STEMI patients without MC. Many of the MC patients sought delayed medical attention, almost half with a latency of more than 24 hours between symptom onset and angiography. Only two patients had a history of PCI or CABG; for the remaining patients, the STEMI leading to the MC was the first manifestation of coronary artery disease. At presentation three out of four patients were in cardiogenic shock. Patients with MC showed more lesions with TIMI 0 or I flow than patients without mechanical complications. Nine patients with ventricular septal rupture, two of them with additional VFWR, were conservatively treated due to futility and died. All three VSR patients, in whom an occluder device was implanted as definite treatment, died within 30 days. The remaining patients with VSR, VFWR and PMR were surgically treated and had 30-day mortality rates of 29%, 50% and 0%, respectively.

The incidence of mechanical complications observed in our cohort of patients admitted due to STEMI is in line with rates previously reported among patients undergoing primary percutaneous coronary intervention; [5–10] however, in contrast to our cohort, populations of these previous studies were largely restricted to patients, who presented within 24 hours of symptom onset.[5, 6, 8] Older age and female sex were associated with the occurrence of mechanical complications after myocardial infarction in previous studies conducted during the thrombolytic and the PCI era.[6, 10–15] The higher percentage of lesions with TIMI 0 or I flow observed in MC patients compared with no-MC patients may reflect the higher risk of developing a mechanical complication in the setting of a transmural infarction.[8, 9] A prolonged latency between symptom onset and hospitalization has been reported to be associated with the occurrence of MC in several studies conducted in the pre-primary PCI era; [16–18] our study supports the continual validity of this association.[10]

The introduction of reperfusion therapies, first thrombolysis, then percutaneous coronary intervention, has considerably decreased mortality after STEMI and the incidence of MC [1, 6, 8, 14, 19]; however, due to the nature of mechanical complications, mortality rates continue to be very high once such a complication develops. [1, 8, 15, 19, 20] This has been substantiated in a recent retrospective study from Spain, which assessed the temporal trends in incidence and outcomes of MC in elderly STEMI patients between 1988 and 2008.[19] Despite a decrease in overall in-hospital mortality rates (from 34.3% to 13.4%) as well as a reduction in the incidence of MC (from 11.1% to 4.3%) over the 30-year observation period, there was neither a significant decline in the proportion of deaths due to MC among all deaths (28.1% to 24.5%), nor the hospital fatality rate of patients suffering a MC (from 87.1% to 82.4%).[19]

Of note is that the proportion of patients undergoing surgical repair in the same study decreased from 45.2% to 17.6% (p = 0.04), with no differences in post-operative survival (28.6% to 33.3%; p = 0.74).[19] In a recent large-scale US study based on administrative data encompassing more than 10,000 patients with VSDs complicating STEMI between 2001 and 2013, the proportion of patients with an attempted corrective procedure was even lower (7.65%); post-procedural mortality rates were high (45.8% after surgery, 76.3% after minimal invasive interventions), particularly in relation to the reported overall in-hospital mortality rate of 30.5%.[15]

Nevertheless, public awareness campaigns to prevent long delays between symptom onset and medical presentation may help to lower the incidence of mechanical complications after myocardial infarction, and rapid echocardiographic evaluation of patients with signs of hemodynamic compromise or cardiac murmurs as well as mechanical circulatory support and subsequent surgical management may increase the chance of survival for individual patients with a MC. [9, 21–24]

The limitations of this study are related to its observational nature, which inherently bears the risk for selection and information biases. Findings of this study only allow inference with regard to STEMI patients surviving until hospital admission; however, a considerable proportion of mechanical complications may occur before presentation or after discharge and, in particular FWR, may lead to immediate death, which precludes identification of the underlying cause in the absence of an autopsy.[11, 25] Survivor bias also limits interpretation of differences in mortality observed in patients treated conservatively, interventionally or surgically. Finally, an assessment of the independent effect of mechanical complications on mortality or a sophisticated statistical evaluation of predictors of mortality within the MC group was precluded by the low event rate.

In this retrospective cohort study conducted in the PCI era, the incidence of mechanical complications after STEMI was low and associated with older age, female sex, and a prolonged latency period between symptom onset and angiography. Overall mortality of MC was high and only MC patients selected to undergo cardiac surgery had a chance of survival, in particular if suffering from papillary muscle rupture.

## Supporting information

S1 Table Mechanical complications: Culprit vessels and frequency of multi-vessel disease. Depicted are counts (percentages). The proportions reported for the culprit vessels refer to the specific complication types. N, number; MVD, multivessel disease. * defined as the presence of coronary stenoses with > 50% luminal narrowing in at least two different coronary arteries as judged by angiography.
(DOCX)

S2 Table Univariable predictors of mortality in the total STEMI cohort. STEMI, ST-elevation myocardial infarction. BMI, body mass index. Hx, past medical history. COPD, chronic obstructive pulmonary disease. MI, myocardial infarction. PCI, percutaneous coronary intervention. CABG, coronary artery bypass grafting. LVEF, left ventricular ejection fraction. LM, left main.
(DOCX)
