# Supplementary material for: Mechanical complications in patients with ST-segment elevation myocardial infarction: A single centre experience
Source: PLoS One. 2019 Feb 22;14(2):e0209502. doi: 10.1371/journal.pone.0209502 (PMC6386360; doi:10.1371/journal.pone.0209502)
Supplement: S1 Table — Depicted are counts (percentages). The proportions reported for the culprit vessels refer to the specific complication types. N, number; MVD, multivessel disease. * defined as the presence of coronary stenoses with > 50% luminal narrowing in at least two different coronary arteries as judged by angiography. (DOCX) [file pone.0209502.s001.docx]

**S1 Table. Mechanical complications: culprit vessels and frequency of multi-vessel disease.**

|  | **N (%)** | **Culprit vessel** | | | **MVD^*^** |
| --- | --- | --- | --- | --- | --- |
|  |  | **LAD** | **LCX** | **RCA** |  |
| **Type:** |  |  |  |  |  |
| Ventricular septal rupture (VSR) | 17 | 8 (47%) | 0 (0%) | 9 (53%) | 13 (76%) |
| Ventricular free wall rupture (VFWR) | 2 | 0 (0%) | 1 (50%) | 1 (50%) | 2 (100%) |
| VSR and VFWR | 2 | 1 (50%) | 0 (%) | 1 (50%) | 2 (100%) |
| Papillary muscle rupture (PMR) | 5 | 0 (0%) | 3 (60%) | 2 (40%) | 3 (60%) |
|  |  |  |  |  |  |
| **Total:** | 26 | 9 (35%) | 4 (15%) | 13 (50%) | 20 (77%) |

Depicted are counts (percentages). The proportions reported for the culprit vessels refer to the specific complication types. N, number; MVD, multivessel disease. ^*^ defined as the presence of coronary stenoses with > 50% luminal narrowing in at least two different coronary arteries as judged by angiography.
