# Supplementary material for: Mechanical complications in patients with ST-segment elevation myocardial infarction: A single centre experience
Source: PLoS One. 2019 Feb 22;14(2):e0209502. doi: 10.1371/journal.pone.0209502 (PMC6386360; doi:10.1371/journal.pone.0209502)
Supplement: S2 Table — STEMI, ST-elevation myocardial infarction. BMI, body mass index. Hx, past medical history. COPD, chronic obstructive pulmonary disease. MI, myocardial infarction. PCI, percutaneous coronary intervention. CABG, coronary artery bypass grafting. LVEF, left ventricular ejection fraction. LM, left main. (DOCX) [file pone.0209502.s002.docx]

**S2 Table. Univariable predictors of mortality in the total STEMI cohort.**

|  | **Sample size** | **Univariable Hazard Ratio (95% CI)** | **p-value** |
| --- | --- | --- | --- |
| **Demographics** |  |  |  |
| Age (per 10 years) | 2508 | 1.72 (1.55-1.91) | < 0.001 |
| Female gender | 2508 | 1.61 (1.23-2.10) | < 0.001 |
| **Comorbidities, lifestyle and medical history** |  |  |  |
| Atrial fibrillation/flutter | 1144 | 1.13 (0.52-2.42) | 0.759 |
| BMI (kg/m^2^) per 10 | 2280 | 0.96 (0.68-1.35) | 0.821 |
| Cerebrovascular accident | 2459 | 2.78 (1.75-4.40) | < 0.001 |
| COPD | 2459 | 2.07 (1.26-3.39) | 0.004 |
| Current Smoker | 2413 | 0.60 (0.44-0.81) | 0.001 |
| Diabetes mellitus | 2465 | 2.32 (1.73-3.10) | < 0.001 |
| Gastrointestinal bleeding | 2459 | 1.97 (0.87-4.43) | 0.102 |
| Hyperlipiemia | 2440 | 0.66 (0.50-0.87) | 0.003 |
| Hypertension | 2453 | 1.38 (1.05-1.82) | 0.020 |
| Peripheral artery disease | 2454 | 2.77 (1.76-4.34) | < 0.001 |
| Previous MI | 2459 | 1.37 (0.89-2.11) | 0.150 |
| Previous PCI | 2459 | 1.39 (0.92-2.10) | 0.113 |
| Previous CABG | 2461 | 2.18 (1.24-3.81) | 0.007 |
| Renal failure | 1209 | 5.19 (3.50-7.68) | < 0.001 |
| History of malignancy | 2457 | 2.79 (1.93-4.04) | < 0.001 |
| **Clinical presentation** |  |  |  |
| Killip III or IV | 2497 | 10.00 (7.77-12.86) | < 0.001 |
| Mechanical complication | 2508 | 9.56 (5.67-16.13) | < 0.001 |
| **Echocardiography** |  |  |  |
| LVEF ≤30% | 2419 | 5.14 (3.92-6.72) | < 0.001 |
| **Laboratory evaluation** |  |  |  |
| Anemia | 1198 | 2.70 (1.89-3.86) | < 0.001 |
| Thrombocytopenia | 1190 | 5.35 (2.36-12.16) | < 0.001 |
| **Procedural factors** |  |  |  |
| Symptom onset to balloon inflation > 24h | 2479 | 0.90 (0.63-1.28) | 0.552 |
| Time from hospital admission to balloon inflation > 60min | 1815 | 0.71 (0.53-0.97) | 0.029 |
| Multivessel treatment (2-3 vessels) | 2495 | 2.23 (1.70-2.92) | < 0.001 |
| Left main LM | 2495 | 4.65 (3.19-6.79) | < 0.001 |
| Total stent length (mm) | 2495 | 1.01 (1.01-1.01) | < 0.001 |
| Mean stent diameter (mm) | 2427 | 0.70 (0.52-0.94) | 0.020 |

STEMI, ST-elevation myocardial infarction. BMI, body mass index. Hx, past medical history. COPD, chronic obstructive pulmonary disease. MI, myocardial infarction. PCI, percutaneous coronary intervention. CABG, coronary artery bypass grafting. LVEF, left ventricular ejection fraction. LM, left main.
